# Forms of Stigma and Discrimination in the Daily Lives of HIV-Positive Individuals in Mauritania

**DOI:** 10.2174/1874613601711010133

**Published:** 2017-12-05

**Authors:** Boushab Mohamed Boushab, Fatim-Zahra Fall-Malick, Mohamed Limame Ould Cheikh Melaïnine, Leonardo Kishi Basco

**Affiliations:** 1 Department of Internal Medicine and Infectious Diseases, Kiffa Regional Hospital, Assaba, Mauritania; 2 National Institute of Hepatitis and Virology, School of Medicine, Nouakchott, Mauritania; 3 Ambulatory Treatment Center, National Hospital Center of Nouakchott, Nouakchott, Mauritania; 4 Research Unit of Emerging Infectious and Tropical Diseases, Institut de Recherche pour le Développement, Aix-Marseille University, Marseille, France

## CORRECTION

The correct name sequence is provided and replaced online which is mentioned as under:

Boushab Mohamed Boushab*, Fatim-Zahra Fall-Malick, Mohamed Limame Ould Cheikh Melaïnine and Leonardo Kishi Basco

The original name sequence was:

Boushab Mohamed Boushab*, Malick Fall Fatim-Zahra, Ould Cheikh Melaïnine Mohamed Limame and Basco Leonardo Kishi

